# Amoxicillin Loaded Chitosan–Alginate Polyelectrolyte Complex Nanoparticles as Mucopenetrating Delivery System for *H. Pylori*

**DOI:** 10.3797/scipharm.1011-05

**Published:** 2011-05-19

**Authors:** Saahil Arora, Sankalp Gupta, Raj K. Narang, Ramji D. Budhiraja

**Affiliations:** Nanomedicine Research Centre, Department of Pharmaceutics, I.S.F. College of Pharmacy, Moga-142001 (Punjab), India

**Keywords:** Factorial design, Mucopenetration, *Helicobacter pylori*, Box Behnken design, Stomach specific delivery system

## Abstract

The present study has been undertaken to apply the concept of nanoparticulate mucopenetrating drug delivery system for complete eradication of *Helicobacter pylori (H. pylori),* colonised deep into the gastric mucosal lining. Most of the existing drug delivery systems have failed on account of either improper mucoadhesion or mucopenetration and no dosage form with dual activity of adhesion and penetration has been designed till date for treating *H. pylori* induced disorders. In the present study, novel chitosan-alginate polyelectrolyte complex (CS-ALG PEC) nanoparticles of amoxicillin have been designed and optimized for various variables such as pH and mixing ratio of polymers, concentrations of polymers, drug and surfactant, using 3^3^ Box-Behnken design. Various studies like particle size, surface charge, percent drug entrapment, *in-vitro* mucoadhesion and *in-vivo* mucopenetration of nanoparticles on rat models were conducted. The optimised FITC labelled CS-ALG PEC nanoparticles have shown comparative low *in-vitro* mucoadhesion with respect to plain chitosan nanoparticles, but excellent mucopenetration and localization as observed with increased fluorescence in gastric mucosa continuously over 6 hours, which clinically can help in eradication of *H. pylori.*

## Introduction

Amoxicillin is a well tolerated, broad-spectrum, beta-lactam antibiotic for the treatment of a wide range of bacterial infections, including *Helicobacter pylori* (*H. pylori*). It inhibits the cell wall biosynthesis during the proliferation phase of *H. pylori* at pH 5 and above, and is first line drug along with Clarithromycin or Metronidazole and omeprazole for the treatment of *H. pylori* induced peptic and duodenal ulcers. Clinical studies using amoxicillin showed least resistance compare to Clarithromycin or Metronidazole against *H. pylori* [1]. Inspite of various antibiotic combinations studied against *H. pylori,* none have shown complete eradication of bacterium. The incomplete eradication of *H. pylori* may be due to sub-bactericidal concentration of antibiotics in the gastric mucosal region, both from the lumen of the stomach and from the gastric supply. Hence local diffusion of drug into gastric mucosa is essential for therapeutic efficacy [2, 3]. The stability of commonly used antibiotics in the triple therapy such as amoxicillin, clarithromycin, or metronidazole is not more than 3–4 hours in gastric environment [4].

Various delivery systems of Amoxicillin have been prepared in recent time for increasing its local availability and efficacy such as polymer matrix tablets [5], gastroretentive floating systems [6–9], bioadhesive systems [10]. Most of these studies emphasised on increasing the retention time of drug in the stomach and increasing the stability of antibiotics in acidic environment of stomach. But these systems could not assist in the complete eradication of bacterium.

Since last decade, the strategy for effective delivery of antibiotics to *H. pylori* has shifted to the use of mucoadhesive micro or nano particulate based delivery systems based on the fact that mucoadhesive particulate show longer retention in stomach and thus deliver the antibiotic locally in the stomach mucosa for longer duration [11]. Most of mucoadhesive systems are prepared out of positively charged polymers such as Chitosan [12, 13], chitosan coated sodium alginate [14], chitosan coated gellan gum [15], Gelatin [16], Carbomer 934P [17], cellulose acetate butyrate (CAB) coated cholestyamine [18], Glidian polymeric protein obtained from gluten [19] etc. These systems provide an intimate contact with the negatively charged (due to sialic acid or carboxyl or sulphate groups in the mucus glycoprotein) mucus membrane due to polyvalent adhesive interaction or electrostatic attraction, H-bond formation, van-der-Waal forces and other [20]. The system has an additional advantage of protecting acid sensitive drugs against acid degradation and offers effective drug diffusion across the mucus layer.

*However H. pylori* has a unique way of survival in harsh acidic environment of the stomach by colonizing deep in the gastric mucosal layer and get adhered to the surface of mucus epithelial cells by adhesion and penetration using flagella [21]. Mucoadhesion though aid in increasing the gastric residence time of particles, the thick viscoelastic mucosal gel does not allow antimicrobial drugs to penetrate through it uniformly [22]. Swelling of the polymer may hinder docking it in gastric mucus and strong mucoadhesion decrease the mobility and thus interpenetrate penetrability in to mucus. In addition, gastric motility and proteolytic activity make mucus turnover intense there by make gastric residence of formulation shorter [23]. Hence efficient adherence to mucus could make the system incapable of penetrating across the mucus layer and entering the underlying epithelia [24].

To overcome these limitations, the particulate system, are required to penetrate the mucus membrane and deliver the drug close proximity to the site of *H. pylori* infection. Many researchers reported the preparation of particulate systems capable of penetrating mucus membrane. Some of these reports include polyethylene glycol (PEG) coated polystyrene based non-adhesive nanoparticle were reported to effectively penetrating sputum of cystic fibrosis patients [25], PEG-PSA(poly sebasic acid) based biodegradable nanoparticles rapidly penetrating human mucus barrier [26], insulin loaded polyethylene glycol-grafted chitosan (PEG-g-chitosan) nanoparticle for the nasal absorption [27], DNA coated biodegradable (poly lactide co-glycolic acid) PLGA nanoparticle for the gene delivery in gastric mucus [28]. These studies emphasized on modifying the surface chemistry of the particulate system such as Chitosan, to minimise the mucoadhesion property by shielding the cationic charge. Along with shielding charge, particle size may also play a very crucial role in the penetration of particle. Particle size less than the mesh size of the mucin fiber are reported to exhibit the good mucin penetration property [25, 27].

The present work is an attempt to develop a novel bi-specific, biodegradable, mucopenetrating chitosan-alginate polyelectrolyte complex (CS-ALG-PEC) nanoparticle system for delivery of Amoxicillin to deep mucus layers near the sanctuary of the *H. pylori.* Cationic polymer, Chitosan was derivatized by the interaction of its surface amino groups with carboxylic acid groups of sodium alginate in absence of Calcium/ magnesium ions which is an unique approach to eradicate *H. pylori* [29].

Various formulation and process variables viz. concentrations and ratio of polymer / drug / surfactant, mixing time and speed, pH, homogenization speed and time etc. influence the characteristics of nanoparticulate delivery systems needing optimization. The concept of mathematical modelling and statistical approach of optimisation, has been considered more powerful than traditional methods of changing one factor at a time for multi factor optimization [30]. The Box-Behnken design (BBD), an independent quadratic design which does not contain any embedded factorial or fractional factorial design is applied here to optimize the process [31–34]. The finally optimized nanoparticles were studied for *in vitro* mucoadhesion and *in vivo* mucopenetration studies on rat model.

## Results and Discussion

### Preparation & optimisation of CS-ALG PEC nanoparticles

The preparation of CS–ALG PEC nanoparticles was based on ionotropic gelation process by mixing aqueous phases of the polycationic CS and polyanionic ALG at room temperature. Due to the higher viscosity of CS solution, a number of experiments were performed by varying the concentration of CS and ALG to screen the appropriate concentration range, pH and mixing ratio of polymeric solutions yielding turbid solution without aggregation.

pH plays an important role in the formation of nanoparticles which affect the size [35]. Preliminary studies were performed by interacting CS and ALG at various pH range (pH 3–6 for CS and pH 3–7 for ALG). The final pH selected for CS and ALG solutions was pH 5.0 and pH 5.5 respectively based on the size of the nanoparticles obtained without aggregation. Increase in pH of CS solution beyond 5.0 showed precipitation particularly when pH is approaching the pKa value of CS (6.5). In addition, pH of CS solution when reduced below 5.0 yielded desecrate particles which were difficult to recover. This is probably due to high degree of protonation of amino group of CS leading to high reaction rate with ALG. [36]. Similar is the case with ALG where the COO^−^ groups affect the formation of nanoparticles, variation in pH from the optimized value (5.5) showed undesirable particle size of the nanoparticles. Rate of addition of ALG into CS solution was also found to a critical parameter in nanoparticle formation [37]. Studies with different mixing ratios of CS: ALG ranging from 1:1 to 10:1 revealed the need for large volume of CS in comparison to ALG. The optimum ratio was found to be 9:1. Larger the volume of CS, more is the spatial interaction between CS and ALG under stirring condition, hence better nucleation. Fluorescent nanoparticles were also prepared in the same way using FITC for visualization.

A direct relationship between the particle size and the CS concentration was observed in pre-optimization studies. Also, the concentration of surfactant (Pluronic F 127) influenced the entrapment efficiency and dissolution of drug. The final concentration range selected for optimization study using Box Behnken design was 0.02–0.06 % w/v of CS, 0.1 % w/v of ALG, 0.01–0.04 % w/v of Amoxicillin and 0.0–0.025 % w/v solution of Pluronic F-127 (surfactant) as tabulated below:

At a concentration of 0.02 % w/v of CS, the particle size of the nanoparticles was found to vary between 264 nm and 321 nm. At 0.04% and 0.06% w/v concentration of CS the size of nanoparticles varied between 382 nm and 600 nm and > 601 nm, respectively and the results are tabulated in Table 2.

Zeta potential less than −30 mV or higher than +30 mV can be an indicator to assure the stability of nanoparticulate systems [38]. Zeta potential of CS ALG PEC nanoparticles depends on the total protonated amino groups on CS. Amino groups interact with acidic groups of ALG for neutralization as described earlier. The zeta potential remained above > + 35 mV at our selected pH range for CS and ALG solutions. This confirms that the system remained stable without aggregation. Also the net positive zeta potential indicates the presence of free surface amino groups on the nanoparticulate delivery system which will help in initial adhesion to gastric mucosa. The observed zeta potential for our prepared nanoparticles was between 35.04 mV and 61.9 mV (Table 2).

The best fit model generated from the software (Design Expert 4.0 trial version) for the observed responses from 15 formulations as per Table 1 (particle size (Y1), zeta potential (Y2) and PDE (Y3)) showed the linear model for particle size and PDE, and quadratic model for zeta potential on the basis of p values (Table 3).

Eq. 1. Particle Size (Y1) =​ 482.64 + 166.87 * A + 42.17 * B + 1.87 * C (Linear Model)

Eq. 2. $$\begin{array}{l} \text{Zeta}\;\text{Potencial}\;(Y2)\;=\;59.68\;+\;3.89\;A-\;2.34\;B\;-\;1.15\;C\;-\;2.32\;A\;B\;-\;0.57\;A\;C\;+\\ 2.73\;*\;B\;*\;C\;-\;4.49\;*\;A^{2}-11.08\;*\;B^{2}\;+\;4.26\;*\;C^{2}\;(\text{Quadratic}) \end{array}$$

Eq. 3. PDE (Y3) = 55.01 * 18.19 * A − 22.35 * B 5.04 * C (Linear Model)

The values of the coefficients for CS, drug and surfactant relates to the effects of the factors - particle size, zeta potential and PDE and their comparative significance of nanoparticulate systems are shown in Table 4.

A direct relationship was observed between the concentration of CS and particle size, zeta potential and PDE. There was a marked increase of all these three parameters when the concentration increased from 0.02–0.06 % (Table 3). It is reported that a synergistic effect exists if the regression equation for a response parameter shows positive value while antagonistic effect in case it is negative [39]. Similarly, the results in Table 3 indicate synergistic interaction between the concentration of CS and surfactant on the responses Y1, Y2 and Y3, whereas concentration of drug showed antagonistic effect on Y3.

**Fig. 1.**
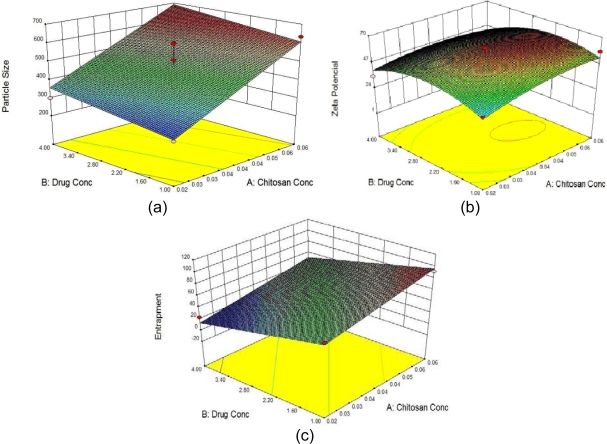
Response surface plot showing effect of Drug and Chitosan concentration on a) Particle size (Y1), b) Zeta potential (Y2) and c) % Drug Entrapment (Y3).

Three-dimensional response surface plots drawn for the graphical optimization of Amoxicillin-loaded CS-ALG PEC mucoadhesive systems are presented in Figure 1.

It was observed that at a constant concentration of surfactant i.e. 0.025% w/v, the PDE of nanoparticles increased with increasing concentrations of CS and decreased with increasing concentration of drug. Higher concentrations of drug resulted in lower PDE and major proportion was washed away in supernatant during separation of nanoparticles.

The optimum formulation of drug-loaded CS-ALG PEC nanoparticles was selected based on the criteria of attaining the maximum value of entrapment efficiency and by applying constraints on Y1 ≥ 600 and Y2 ≥ +35 mV (Table 1). The formulation composition with CS 0.06%, drug 0.01% and Pluronic F-127 (surfactant) 0.019% w/v was found to fulfil requisites of an optimum formulation. The optimized formulation with the particle size, zeta potential and the PDE as 651 nm, +59.76 mV and 91.23% respectively has been used for the rest of the study.

For all of the three checkpoint formulations, the results of the evaluation for particle size, zeta potential and entrapment efficiency were found to be within the 95% confidence interval limits (Table 5).

**Fig. 2.**
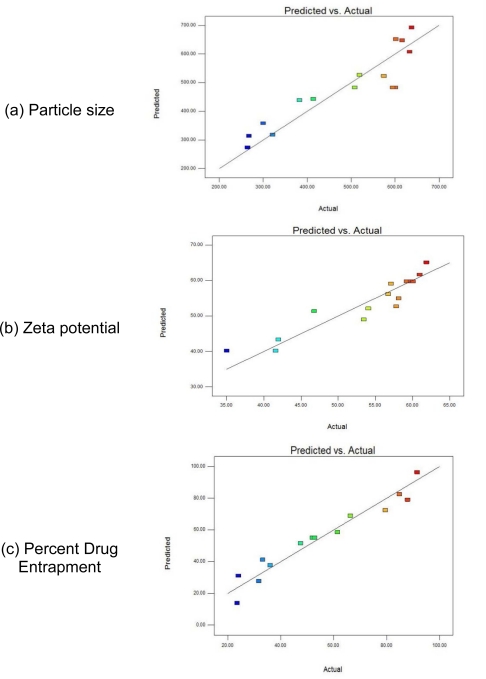
Linear correlation plots between actual and predicted values of (a) Particle size; (b) Zeta potential; (c) Percent Drug Entrapment.

Percentage prediction error helped in the validation of generated regression equations. Linear correlation plots between the actual and the predicted response variables (Figure 2) showed the scatter of the residuals versus actual values for better representing the spread of the dependent variables under present experimental settings. For validation of RSM results, the experimental values of the responses were compared with that of the anticipated values and the prediction error for the three response variables were found to vary between −8.16% and +16.28%. The low magnitudes of errors as well as the significant values of correlation validated the proposed RSM and proved the high prognostic ability of the Box-Behnken designs in formulation of nanoparticles.

### Stability of Amoxicillin in simulated gastric fluid (pH 1.2)

**Fig. 3.**
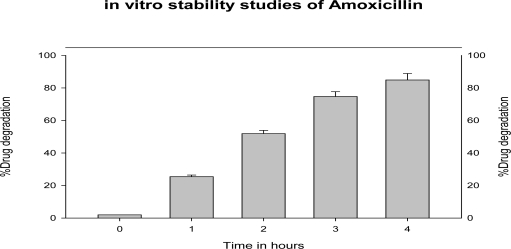
*In vitro* stability of amoxicillin in simulated gastric fluid (pH 1.2) (n = 3).

The drug stability studies in SGF (pH1.2) showed that the drug degraded up to 85% (n=3) in acidic environment of the stomach in 4 hrs as depicted (Figure 3). Similar studies performed on CS-ALG PEC nanoparticles showed the decreased degradation of drug. The amoxicillin-loaded CS–ALG PEC nanoparticles showed 50%, 67% and 76 % release of amoxicillin at 2, 4 and 6 hours, respectively, which indicated the protective efficiency of nanoparticulate delivery system in gastric environment even after 6hrs. This protective behaviour can be attributed to the existence of amoxicillin in inner non-hydrated part of nanoparticles as in other matrix systems. Thus CS-ALG PEC nanoparticles can be utilised as sustained release gastroretentive delivery system for antibiotics like Amoxicillin, Clarithromycin or Metronidazole in eradication of *H. pylori* where antibiotic formulations fail to deliver the minimum inhibitory concentration in gastric mucosa due to instability at low pH & short residence time in the stomach [12].

### Characterization of Nanoparticles:

**Fig. 4.**
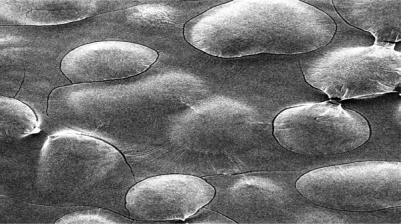
SEM micrograph of amoxicillin loaded CS-ALG polyanionic nanoparticles

The Scanning electron micrographs of freeze dried optimised amoxicillin loaded CS-ALG PEC nanoparticles at 200X magnification are showed in figure 4. The nanoparticles were seen as distinct spherical, consistent solid surface with porous structure. The observed microporous matrix structures of polyelectrolyte complex can be formed due to electrostatic interactions between anionic groups from sodium alginate and cationic groups from CS. [40]

**Fig. 5.**
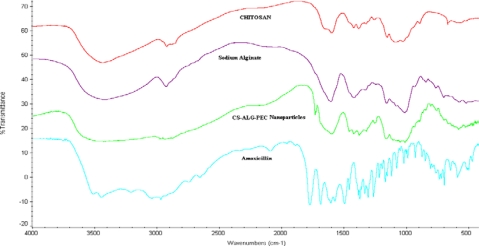
FTIR spectra of CS-ALG PEC nanoparticles with Amoxicillin

CS, ALG and CS–ALG PEC nanoparticles were analysed using FT-IR spectrophotometer for characteristic absorption bands, indicative of their interaction as shown in Figure 5. The peak at ∼1640 cm^−1^ in both the CS and CS–ALG PEC nanoparticles spectra was due to the unreacted NH_2_-groups of CS. Similarly, peaks observed at ∼820 cm^−1^ and ∼1320 cm^−1^ in FT-IR spectra of ALG and CS–ALG PEC nanoparticles represent unreacted -COOH groups of ALG. The characteristic peak observed at 1447 cm^−1^ (salt of carboxyl group) in the FT-IR spectrum of nanoparticles was attributed to the ionic interaction between these two reactive groups [41].

### Drug release profile

**Fig. 6.**
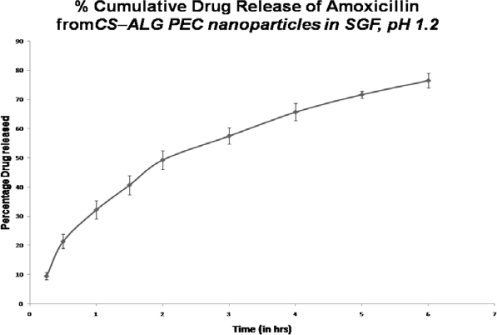
*In vitro* release profiles of the optimised formulation of CS–ALG PEC nanoparticles in SGF, pH 1.2

The in vitro drug release study of the optimized formulation in SGF (pH 1.2) showed about 76% of release of Amoxicillin over a period of 6 h. (Figure 6) As shown, a fast release of drug (∼49%) is observed in first two hours which is further sustained 57.5%, 65.7% & 76.5% in 3^rd^, 4^th^ & 6^th^ hour respectively. The % drug release data from the mucoadhesive CS–ALG PEC nanoparticles followed the Higuchi model (k=32.42h^−1^ r^2^ = 0.9905). By applying the % drug release data in Korsmayer or power model to understand the release mechanism, the release exponent ‘n’ value was found out as 0.618, which indicates non-Fickian (anomalous) release. This can be attributed to presence of unbound drug on the surface of nanoparticles or high swelling degree of these poly electrolyte complexes.[19] The release mechanism of Amoxicillin from the CS-ALG PEC nanoparticles refers to a combination of both diffusion and erosion controlled drug release, which is attributed to the rapid hydration of CS and ALG resulting in swelling & erosion of poly-ionic complexes. The observed release mechanism by diffusion would be useful in stomach specific delivery systems. [40].

### In-vitro mucoadhesion studies

The Bioadhesive force studies on optimised CS-ALG PEC nanoparticles revealed the detachment stress up to 14.98 × 10^3^ dyne/cm^2^ (n=3). The *in vitro* mucoadhesion studies using the FITC-labelled CS-ALG PEC nanoparticles and FITC-labelled Chitosan-Tripolyphosphate (TPP) nanoparticles showed mucoadhesive capacity with a percent mucoadhesion of 75.94 ± 3.2% and 88.5 ± 6.2% (n=3), respectively. The cationic amino groups present on the chitosan interact electrostatically with mucin glycoproteins, sialic acid and other anionic moieties present on gastric mucosa [42]. The decrease in mucoadhesive capacity of prepared CS-ALG PEC nanoparticles can be attributed to decrease in surface amino groups by ionic interaction with carboxylic ions of sodium alginate [24]. This decline in mucoadhesion can help the nanoparticles to infiltrate at faster rate in gastric mucosa thus proving the utility of prepared CS-ALG PEC nanoparticles better for better penetration and accumulation at the site of *H. pylori* infection beneath mucosa.

### In-vivo mucopenetration studies

**Fig. 7.**
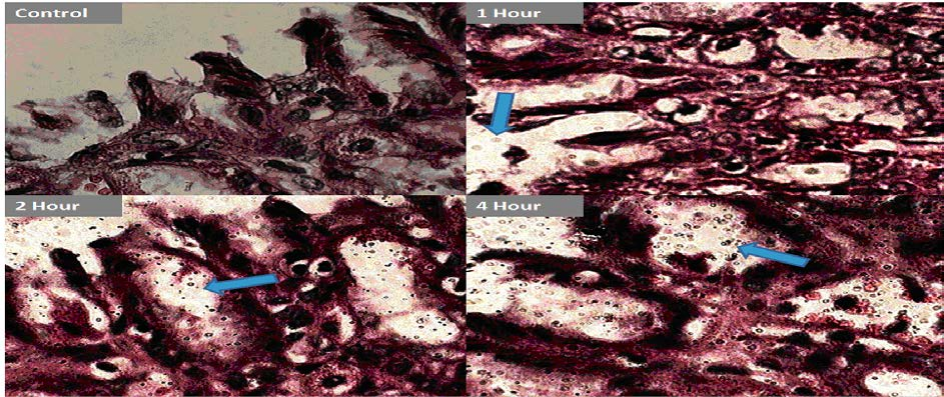
*In-vivo* mucopenetration studies of CS-ALG PEC on Gastric mucosa (Digital microscope magnification-100X)

**Fig. 8.**
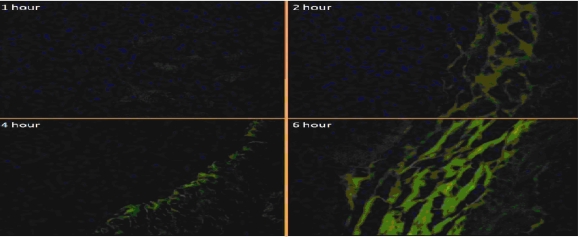
*In-vivo* mucopenetration studies of CS-ALG PEC on Gastric mucosa (Fluorescent microscope magnification-40X)

The microscopic studies revealed the mucopenetration as well as localisation of quite good number of FITC labelled CS-ALG PEC nanoparticles into the gastric mucosa during 6 hours study. (Figure 7, 8).

The amoxicillin loaded FITC labelled CS-ALG PEC nanoparticles have shown good initial gastric mucoadhesion and finally penetrated deep in to the mucosal layers near the gastric epithelial cells of antrum region with time, as observed with increased fluorescence in this site of mucosa continuously over 6 hours. (Fig. 8) The observed mucopenetration is attributed to decrease in surface positivity & hence mucoadhesion of CS-ALG PEC nanoparticles in comparison to plain chitosan nanoparticles. Thus the results confirm the earlier studies on modifying the surface chemistry &/or shielding or decreasing the cationic charge on polymers like chitosan in order to increase the motility in mucosa [25–27]. These studies indicate the possible improvement in the efficacy of CS-ALG PEC nanoparticles and may significantly reduce the chances of incomplete eradication and systemic side effects.

## Conclusion

A novel mucopenetrating CS-ALG PEC nanoparticulate system composed of chitosan and sodium alginate was successfully optimized using 3^3^ Box-Behnken design of experimentation. The optimum complexation was found using chitosan at pH 5 and sodium alginate at pH 5.5 and the most effective composition of CS-ALG PEC nanoparticles was chitosan 0.06 % w/v, Amoxicillin (drug) 0.01 % w/v and Pluronic F-127 (surfactant) 0.019 % w/v. The *in vitro* drug release studies for 6 hrs revealed the gastro protective nature of PEC system and diffusion through swollen polymeric complex as main drug release mechanism.(Higuchi model) The *in vitro* mucoadhesion studies (76 % mucoadhesion) confirmed the decrease in mucoadhesion of chitosan by ionic interaction with anionic sodium alginate. The *in vivo* mucopenetration studies using Fluorescent FITC labelled CS-ALG PEC nanoparticles showed increased intensity of fluorescence near the gastric epithelial layers confirming mucopenetration as well as localization of nanoparticles in deep mucosal region. The results proved the concept of increased mobility of nanoparticles in the gastric mucus by decreasing the surface amino groups of CS on ionic interaction with carboxylic groups of ALG. Hence the current novel CS-ALG PEC nanoparticles can be utilised for suggested transmucosal delivery of antibacterial drugs in eradication of *H. pylori.*

## Experimental

### Materials

Chitosan (CS) (viscosity 200–400 mPas), Pluronic F-127 (cell culture tested) and Fluorescein isothiocynate isomer l-Celite (FITC) were purchased from Sigma Aldrich (USA). Sodium Alginate (ALG) and D-(+)-Trehalose dihydrate were purchased from HiMedia Laboratories Pvt. Ltd (Mumbai). Amoxicillin trihydrate was provided as a gift sample from Siemens Laboratories (India) Gurgaon. Simulated gastric fluid (SGF) was prepared as per USP XXIX. Double distilled water was used in all the preparations. All other solvents and chemicals used were of analytical grade.

### Preparation of CS-ALG PEC nanoparticles

The nanoparticles were prepared by modified ionic gelation method [35]. A mixture of chitosan, pluronic and amoxicillin was prepared in varying concentrations of all the components. Chitosan solution (0.02–0.06 % w/v) was prepared in 1 % v/v acetic acid (pH adjusted to 5 using 1 M NaOH). To this was added Pluronic F 127 (0.0–0.025 % w/v) and amoxicillin trihydrate (0.01–0.04 % w/v). To the above mixture, 0.1 % w/v aqueous solution of ALG (prepared in double distilled water, pH was adjusted to 5.0 using 0.05 M HCl) was sprayed with continuous stirring for 30 minutes [36, 43]. The nanoparticles produced were collected by centrifugation at 25,000 rpm (42,000 g) for 50 minutes, washed with double distilled water and freeze dried (pre-freezing at −20°C in deep freezer) (Martin Christ model Alpha 1–2 LD *plus*) at −55°C, 0.01mm of Hg using D-(+)-Trehalose, dihydrate (0.5 % w/v) as a cryoprotectant.

### Preparation of Fluorescent CS-ALG PEC nanoparticles

FITC conjugated Chitosan was prepared by allowing it to react with CS. FITC in methanol was prepared as a solution and added into 1 % solution of CS slowly [44]. The reaction was allowed to proceed in dark at room temperature for 2 hours. The resultant product was precipitated using 0.1 M sodium hydroxide solution, washed extensively with double distilled water, until the wash was freed from FITC fluorescence signal. The precipitate (FITC labelled chitosan) was freeze dried.

The FITC labelled CS-ALG nanoparticles were prepared as per the method described earlier.

### Optimization, data analysis and model validation of drug loaded CS-ALG PEC nanoparticles (Box-Behnken Design)

The selected method for preparation of nanoparticles involve several formulation variables such as pH, concentration of ingredients, stirring speed, mixing volume etc which influences the particle size, surface charge on the particles, entrapment efficiency and release profile of the nanoparticles. Moreover, there are chances of interactions between the various variables which may alter the above mentioned characteristics. Hence, a need arose for optimization of the nanoparticle preparation using a suitable design which could reduce the number of experimentation protocols. Box–Behnken design fit the requisite need of reducing experimentation protocols because 3–factor, 3–level of experimentation reduced the total number of experiments to just 15 instead of 27 as per conventional optimization techniques [45]. Design Expert software (Version 8.0.1, Stat-Ease) was used to explore the response surfaces and to construct second order polynomial models [46]. High drug entrapment and stability with optimum particle size are essential features for nanoparticulate delivery systems. Hence, for the present study, constraints like particle size (≥600 nm), zeta potential (≥ +35 mV) and maximum percent drug entrapment (PDE) were fixed.

A design matrix comprising of fifteen experimental runs was constructed, for which the non-linear computer generated quadratic model is defined as;

Eq. 4. $$Y=b_{0}+b_{1}A+b_{2}B+b_{3}C+b_{12}A*B+b_{13}A*C+b_{23}B*C+b_{11}A^{2}+b_{22}B^{2}+b_{33}C^{2}$$

Where, Y = measured response associated with each factor level combination; b_0_ = intercept; b_1_ to b_33_ are regression coefficients computed from the observed experimental values of Y from experimental runs; and A, B and C are the coded levels of independent variables [47, 48]. Table 1 lists the independent variables studied i.e. concentration of CS (A), Amoxicillin (B) and Pluronic F-127 (C) in the formulation, along with their levels, selected on the basis of preliminary experimentation [49]. The designed fifteen experimental formulations, with respective concentrations of formulation variables and the corresponding observations for dependent variables like particle size (Y_1_), zeta potential (Y_2_) and PDE (Y_3_) are given in Table 2. The responses obtained were fitted to first order, second order and quadratic-models and evaluated for statistical significance and r^2^ values. Three-dimensional response surface plots were generated by Design Expert 8.0.1 software. Finally, three optimal checkpoint formulations were selected to validate the chosen experimental domain and polynomial equations. The resultant experimental values of the responses were statistically compared and validated [46].

### Stability of Amoxicillin in simulated gastric fluid (pH 1.2)

10 mg of amoxicillin was dissolved in simulated gastric fluid (pH 1.2) in a 50ml volumetric flask and maintained at 37°C [40]. The % degradation of the drug in SGF was determined at 0, 1, 2, 3, 4 and 6 hours, spectrophotometrically, at 272.6 nm.

### Characterization of Nanoparticles

#### Percentage yield

The unentrapped drug from drug loaded CS-ALG PEC nanoparticles was removed by washing with distilled water (thrice). Washed nanoparticles were re-dispersed in distilled water and subjected to lyophilisation. Lyophilized nanoparticles were analyzed for yield [48].

The nanoparticles yield was calculated using the following equation:

Eq. 5. $$\%\;\text{Yield}\;\text{of}\;\text{Nanoparticles}=\frac{\text{Mass}\;\text{of}\;\text{nanoparticles}\;\text{recoverd}}{\text{Mass}\;\text{of}\;\text{all}\;\text{ingredients}}\times 100$$

#### Entrapment efficiency (PDE)

The percent of drug entrapment of drug loaded nanoparticles were calculated as below:

Nanoparticles were digested in acetic acid for 20 minutes and centrifuged at 1000 rpm for 5 minutes. The supernatant was withdrawn, filtered and estimated for drug content [29]. The % drug entrapment was calculated using the following equation:

Eq. 6. $$\%\;\text{Entrapment}\;(\text{PDE})=\frac{\text{Amount}\;\text{of}\;\text{drug}\;\text{present}\;\text{in}\;\text{nanoparticles}}{\text{Total}\;\text{amount}\;\text{of}\;\text{drug}\;\text{added}}\times 100$$

#### Drug release profile

Drug loaded CS-ALG PEC nanoparticles were evaluated for release in SGF (pH 1.2). Accurately weighed nanoparticles were dispersed in water and kept in a pre-treated dialysis tube membrane in SGF (pH 1.2) at 37 ± 1°C with continuous stirring. Aliquots were withdrawn at 0.25, 0.5, 1.0, 1.5, 2.0, 2.5, 3.0, 4.0, 5.0 and 6.0 hours, filtered through 0.22 μm membrane filter and analysed for drug content [48].

#### Particle size and Zeta potential

Size distribution and zeta potential of drug loaded CS-ALG PEC nanoparticles were done using Zetasizer (Beckman Coulter, Delsa nano C). All measurements were obtained in triplicate (n = 3) [18].

#### Surface Morphology

Drug loaded CS-ALG PEC nanoparticles were subjected to surface electron microscopy (SEM, ZEISS EVO 50) [51]. Freeze dried nanoparticles were mounted on aluminium sample holder and gold coated for morphological analysis with an applied voltage of 20 kV at various magnifications.

### Polymer–Drug / polymer–polymer interaction studies

FTIR (Thermo nicolet-380) was performed on freeze dried CS-AlG PEC nanoparticles to assess the interaction between amino groups of CS and carboxylic groups of ALG using potassium bromide (KBr) discs, compressed at 100kg/cm^2^ with an hydraulic pellet press and scanned at 4 mm/s at a resolution of 2 cm over a wave number region of 400–4000 cm^−1^.

### Mucoadhesion & Mucopenetration studies

#### (a) Bioadhesion Force studies

Drug loaded CS-ALG PEC nanoparticles were subjected for studies to calculate the force required to detach from gastric mucosal tissue as per the modified method of ElHady SSA [52] using wistar rats.

#### (b) In-vitro mucoadhesion studies

The percentage mucoadhesion was calculated by count number method using Neubauer’s chamber (haemocytometer) [53]. The previously counted number of FITC-labelled CS-ALG PEC nanoparticles (n_1_) (10 mg of nanoparticles dispersed in 2 ml of double distilled water) were filled in the pre-excised & washed isolated rat stomach, closed from one end. After 20 minutes of incubation at 37°C, stomach was washed with 0.9%w/v NaCl solution (thrice) and the washings were collected. These washings were analysed for total nanoparticles count (n_2_) under gamma effect of Motic digital microscope in 100 X. Similar studies were performed using FITC-labelled CS-TPP nanoparticles (using tripolyphosphate anions (TPP) as cross linking agent) for comparative evaluation of mucoadhesion.

#### (c) in-vivo mucopenetration studies

The animal studies experimental protocol was approved by the Institutional Animal Ethics Committee (IAEC) in accordance with Committee for the Purpose of Control and Supervision of Experiments on Animals (India) (CPSCEA) guidelines. Twelve healthy male Wistar rats (170–230 g), fasted overnight, subjected to standard laboratory conditions (i.e. room temperature, 23 ± 2°C; relative humidity, 50 ± 5%; 12/12 hours light/dark cycle), were administered with 10 mg / 2 ml of FITC labelled CS-ALG PEC nanoparticles using oral feeding canula. The animals were sacrificed at time intervals of 1, 2, 4 and 6 hours. The stomach was excised, washed (0.9%w/v NaCl solution), fixed (antrum region) in 10 % formalin, sectioned to a thickness of 5micronm and stained with eosin. [47] The fixed stained tissue sections were evaluated under Digital microscope (100X) (Motic DMWB series) and inverted fluorescent microscope 40X (Olympus) to analyse the mucopenetration and localization of fluorescent CS-ALG PEC nanoparticles.

## Figures and Tables

**Tab. 1.** Variables in Box–Behnken Design

**Factors**	**Units**	**Levels used actually (coded)**		
		**Low (−1)**	**Medium (0)**	**High (1)**

Chitosan Concentration	%w/v	0.02	0.040	0.060
Drug Concentration	%w/v	0.01	0.025	0.040
Surfactant Concentration	%w/v	0.00	0.012	0.025

**Dependent Variables**	**Units**	**Constraints**		

Y1 = Particle Size	nm	Y1 ≥ 600		
Y2 = Zeta Potential	mV	Y2 ≥ 35.04		
Y3 = PDE	%	Maximize		

**Tab. 2.** Observed responses for Box-Behnken design for CS-ALG PEC nanoparticles

**Formulation Code**	**Chitosan (% w/v)**	**Drug (%w/v)**	**Surfactant (%)**	**Particle Size (Y1) (nm)**	**Zeta Potential (Y2)(mV)**	**PDE (%) (Y3)**
F1	0.02	0.01	0.012	265±16	41.59±3.9	61.5±4.6
F2	0.06	0.01	0.012	633±23	57.81±3.1	91.6±4.0
F3	0.02	0.04	0.012	300±19	35.04±2.4	23.5±3.1
F4	0.06	0.04	0.012	638±27	41.97±2.8	47.5±4.3
F5	0.02	0.025	0.000	268±18	56.75±3.7	24.0±2.9
F6	0.06	0.025	0.000	616±24	61.90±4.1	66.4±2.4
F7	0.02	0.025	0.025	321±21	58.15±3.9	33.2±3.6
F8	0.06	0.025	0.025	601±23	60.99±2.6	88.0±3.5
F9	0.04	0.01	0.000	382±17	57.12±3.5	79.6±2.8
F10	0.04	0.04	0.000	574±25	53.46±3.2	31.8±4.1
F11	0.04	0.01	0.025	414±22	46.79±3.3	84.9±4.3
F12	0.04	0.04	0.025	519±29	54.08±4.6	36.0±4.5
F13	0.04	0.025	0.012	600±18	59.22±4.2	52.4±2.9
F14	0.04	0.025	0.012	508±18	59.76±2.9	52.0±3.8
F15	0.04	0.025	0.012	594±21	60.06±3.6	52.8±4.7

**Tab. 3.** Summary of Results describing Regression Analysis for responses Y_1_, Y_2_ and Y_3_

**Models**	**Sequential p-Value**	**R^2^ Value**	**Adjusted R^2^**	**Predicted R^2^**	**% C.V.**	**Remarks**
**Response (Y1)**						
Linear model	0.0001	0.8362	0.7915	0.7447	13.46	Suggested
Second order	0.8927	0.8477	0.7334	0.5905	15.22	
Quadratic model	0.1224	0.9477	0.8534	0.4199	11.29	

**Response (Y2)**						
Linear model	0.5116	0.1820	−0.0411	−0.6391	15.8	
Second order	0.8994	0.2367	−0.3357	−2.5908	17.9	
Quadratic model	0.0234	0.8688	0.6327	−1.0937	9.38	Suggested

**Response (Y3)**						
Linear model	< 0.0001	0.9409	0.9248	0.8762	11.54	Suggested
Second order	0.8083	0.9473	0.907817	0.731027	12.78	
Quadratic model	0.7467	0.9579	0.882211	0.327505	14.45	

Regression equations of the fitted models

Y = b_0_ + b_1_A + b_2_B + b_3_C + b_12_AB + b_13_AC + b_23_BC + b_11_A^2^ + b_22_B^2^ + b_33_C^2^

**Tab. 4.** Coefficients for the particle size, zeta potential and % Entrapment efficiency

**Term**	**Particle size (nm)**			**Zeta potencial (mV)**			**PDE**		

	**Coeff.**	**SE**	Range^*^	**Coeff.**	**SE**	Range^*^	**Coeff.**	**SE**	Range^*^
Intercept	482.64	16.78	445.72 to 519.57	59.68	2.91	52.21 to 67.15	55.01	1.64	51.40 to 58.62
A-Chitosan Conc.	166.88	22.97	116.32 to 217.43	3.89	1.78	−0.68 to 8.47	18.91	2.24	13.97 to 23.85
B-Drug Conc.	42.18	22.97	−8.30 to 92.73	−2.35	1.78	−6.92 to 2.23	−22.36	2.24	−27.30 to 17.42
C-Surfactant Conc.	1.88	22.97	−48.68 to 52.43	−1.15	1.78	−5.73 to 3.42	5.04	2.24	0.10 to 9.98
AB	–	–	–	−2.32	2.52	−8.79 to 4.15	–	–	–
AC	–	–	–	−0.58	2.52	−7.05 to 5.89	–	–	–
BC	–	–	–	2.74	2.52	−3.73 to 9.21	–	–	–
A^2^	–	–	–	−4.50	2.62	−11.23 to 2.24	–	–	–
B^2^	–	–	–	−11.08	2.62	−17.82 to −4.35	–	–	–
C^2^	–	–	–	4.26	2.62	−2.47 to 11.00	–	–	–

* The range indicates the lower and upper value of coefficients at 95% confidence interval.

**Tab. 5.** Composition of Checkpoint formulations, predicted and experimental values of response variables and percentage prediction error with 95% Confidence Interval.

**Optimized formulation**	**Response Variable**	**Predicted value**	**Experimental value**	**Percentage prediction error**	**95% Confidence Interval**
0.06:0.01:0.019	Y1	600	638.1	6.35	516.80 to 683.10
	Y2	51.65	60.06	16.28	40.02 to 63.23
	Y3	98.28	91.23	−7.17	89.82 to 106.00

0.06:0.01:0.020	Y1	600	601.7	0.28	516.80 to 683.10
	Y2	51.63	59.76	15.75	40.01 to 63.21
	Y3	97.94	89.95	−8.16	89.59 to 105.70

0.06:0.01:0.018	Y1	599.61	616.4	2.80	516.10 to 682.80
	Y2	51.66	59.22	14.63	40.05 to 63.26
	Y3	97.51	91.84	−5.81	89.22 to 105.54
